# Stability of high-mass molecular libraries: the role of the oligoporphyrin core

**DOI:** 10.1002/jms.3526

**Published:** 2015-01-19

**Authors:** Uĝur Sezer, Philipp Schmid, Lukas Felix, Marcel Mayor, Markus Arndt

**Affiliations:** a University of Vienna, Faculty of Physics, VCQ and QuNaBioSBoltzmanngasse 5, 1090, Vienna, Austria; b Department of Chemistry, University of BaselSt. Johannsring 19, 4056, Basel, Switzerland; c Karlsruhe Institute of Technology (KIT), Institute of NanotechnologyP.O. Box 3640, 76021, Karlsruhe

**Keywords:** macromolecular beams, laser desorption, photoionization, TOF-MS, molecular libraries

## Abstract

Molecular beam techniques are a key to many experiments in physical chemistry and quantum optics. In particular, advanced matter-wave experiments with high-mass molecules profit from the availability of slow, neutral and mass-selected molecular beams that are sufficiently stable to remain intact during laser heating and photoionization mass spectrometry. We present experiments on the photostability with molecular libraries of tailored oligoporphyrins with masses up to 25 000 Da. We compare two fluoroalkylsulfanyl-functionalized libraries based on two different molecular cores that offer the same number of anchor points for functionalization but differ in their geometry and electronic properties. A pentaporphyrin core stabilizes a library of chemically well-defined molecules with more than 1600 atoms. They can be neutrally desorbed with velocities as low as 20 m/s and efficiently analyzed in photoionization mass spectrometry. Copyright © 2015 John Wiley & Sons, Ltd.

## Introduction

In recent years, advanced chemical synthesis and mass spectrometry have become enabling tools for matter-wave experiments with complex molecules, and quantum interferometry was able to provide new insights into molecular properties, structure or dynamics.[1,2] The partnership between these disciplines has recently led to the development of new matter-wave interferometers. In particular, a Kapitza–Dirac–Talbot–Lau interferometer was successfully used for demonstrating the quantum wave nature of entire macromolecules with masses in excess of *m* = 10 000 Da.[3] Complementary to that, a matter-wave interferometer with three pulsed laser light gratings (OTIMA) was established to visualize quantum delocalization even of clusters of molecules, for de Broglie wavelengths as small as *λ*_dB_ ≃ 200 fm.[4] Both devices were built to explore the limits of quantum physics in the regime of high masses[2] and to enable new measurements on neutral nanoparticles.[2,5–8] Here, we focus on new experiments with fluoroalkylsulfanyl-functionalized oligoporphyrins, two molecular libraries based on two cores of different aromaticity. We study their structural stability under laser desorption and photoionization up to masses around 25 000 Da.

Several methods have been developed to launch massive and fragile molecules: electrospray ionization[9] (ESI) and matrix-assisted laser desorption ionization[10] (MALDI) are soft-volatization techniques for particles up to the MDa mass range.[11] However, ESI produces mainly highly charged ions and is initially accompanied by a dense carrier gas. In contrast to that, MALDI is typically operated under high vacuum, and the molecules are released carrying none or only a few charges.[12] MALDI often uses an organic matrix, such as dihydroxybenzoic acid, to enhance absorption of the desorbing UV laser and to assist in charging the analytes. Moreover, without the acid matrix environment, proton exchange in the sample is reduced. We exploit this to generate *neutral* molecules. The analyte itself can act as its own matrix if it is sufficiently absorptive and photostable. The resulting beam velocities can be smaller by an order of magnitude than in common MALDI studies.[13,14]The desorption of neutral molecules can be monitored by post-ionization mass spectrometry. In earlier studies, photoionization of organic molecules was typically limited to particles below 2000 Da.[15–17] Large clusters of guanine, tryptophan and gramicidin could still be observed using single-photon ionization[18,19] – with masses up to 7000 Da.

Molecules can also be tailored to facilitate volatilization and photoionization. The covalent tagging of biomolecules with ionizable chromophores has already been implemented before,[20–24] and also the functionalization of a chromophore *monomer* with long fluoroalkylsulfanyl chains[25,26] was successful. Here, we extend this concept by exploring different molecular *oligomers* in the attempt to enhance the molecular stability at even higher mass.

## Functionalization of two oligoporphyrins

**Figure 1:**
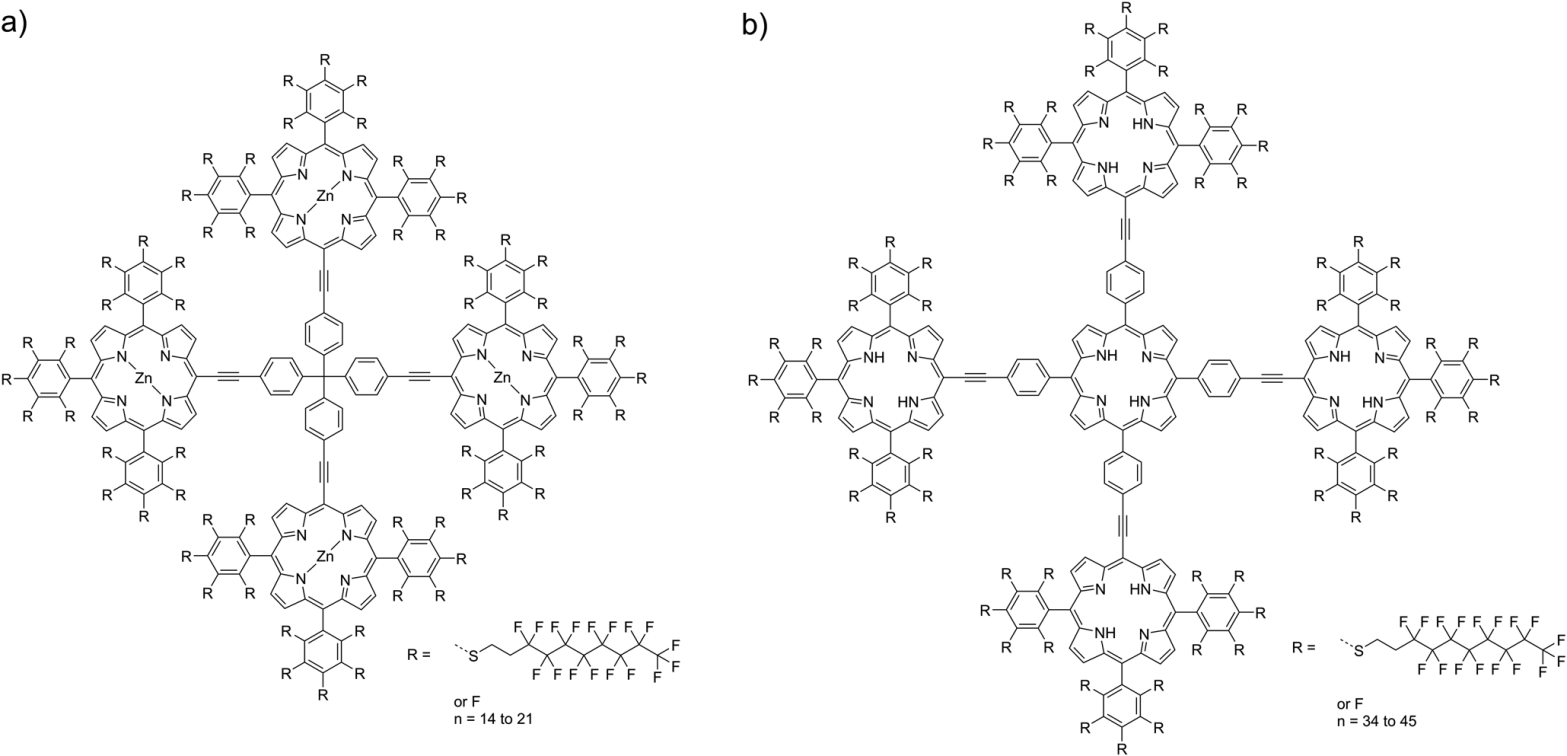
Molecular library 1 (a) consists of four porphyrin systems with a central metal atom (Zn). They are linked via a tetraphenylmethane unit, which breaks the conjugation between the porphyrins. The most abundant molecule has *n* = 17 chains and a mass of 11 676 Da. Library 2 (b) is based on a metal-free porphyrin pentamer that is fully conjugated in the core. The most abundant molecule has *n* = 40 chains and a mass of 22 152 Da.

Applications in quantum interferometry will profit from a molecular library, i.e. a molecular ensemble with well-defined mass separation.[3,4] At a fixed molecular beam velocity, mass variation implies a controlled tuning of de Broglie wavelengths (*λ*_dB_ = h/mv). We compare two molecular libraries[27] as shown in Fig.1: a tetrahedral arrangement of four zinc-coordinated porphyrins arranged around one tetraphenylmethane core and a planar pentaporphyrin. The pentaporphyrin is expected to reduce its ionization energy, and the steric configuration is supposed to facilitate the escape of ejected electrons upon ionization. Both oligomers are functionalized by different numbers of fluoroalkylsulfanyl chains to boost the molecular mass while keeping the electronic polarizability low. The ionization energy of each porphyrin is smaller than 7 eV.[28] The central oligomer therefore increases both the number of functional anchor points and the photoionization cross section for vacuum ultraviolet (VUV) light.[29]

## Experimental design

**Figure 2:**
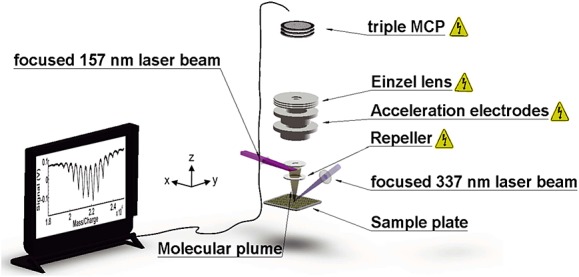
Experimental scheme for short-pulse laser desorption and post-ionization. The focused 337-nm beam of an N_2_ laser (waist *w_x_* = 1.5 mm, *w_y_* = 0.8 mm) releases a molecular plume that expands into the ion extraction region. A 157-nm laser beam (39 mm further above) intersects with the molecules and ionizes them. The cations are detected using time-of-flight mass spectrometry (TOF-MS).

In order to assess the VUV photoionization properties of library **1** and **2**, we devised an experiment as shown in Fig.2. We dissolve 2.5 mg of library **1** in 1.5 ml of pure diethyl ether or 7.5 mg of library **2** in 1.5 ml pure *tert*-butyl methyl ether. Droplets of this solution *without* any matrix are deposited on a clean stainless steel plate and dried in air. The N_2_ laser beam (*E* = 90 μJ, *τ* = 3 ns) is focused onto the sample plate under an angle of 30°. During desorption, the sample plate is laterally shifted to ensure that each laser shot hits a fresh sample spot.

The neutral molecular beam is ionized by the VUV light of an F_2_ excimer laser beam (*λ* = 157.6 nm, *τ* = 8 ns, *E* < 1.4 mJ), focussed into a rectangular shape of 8 ± 1 mm^2^. The ions are detected by a linear time-of-flight mass spectrometer (Kaesdorf, *m*/Δ*m* ≈ 100) in Wiley McLaren[30] configuration.

## Mass spectra

**Figure 3:**
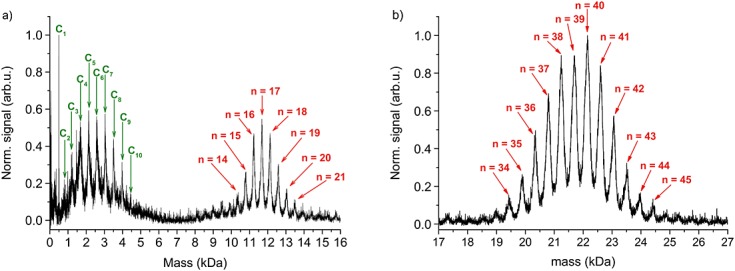
Mass spectrum of the post-ionized molecular library 1 (a) and library 2 (b). The excimer laser was set to 1.4 mJ/pulse, and the delay to the desorption pulse was set to the maximum of the velocity distribution of the neutral molecules. In panel (a), the most significant peak at *n* = 17 corresponds to a mass of 11 676 Da. In panel (b), the maximum at *n* = 40 corresponds to 22 152 Da. Distinct peaks are still observed beyond 25 kDa.

Figure3 shows the post-ionization mass spectra of the two libraries. Each scan was averaged over 30 laser shots. The desorption energy was identical for both spectra. The spectrum of molecular library **1** consists of eight well-resolved peaks (Fig.3(a)), each representing a different number *n* of fluoroalkylsulfanyl chains, where *n* varies by 3–4 around the most probable value *n* = 17. A molecule with 21 chains corresponds to a mass of 13 492 Da. The mass difference between neighbouring peaks is 450 ± 10 Da, resulting from the nominal mass of a single chain (479 Da) minus the fluorine atom that is substituted in order to attach the chain. The characteristic abundance of all peaks in this mass spectrum agrees well with MALDI spectra.

The post-ionization mass spectrum also shows a distribution of low masses, the most prominent one denoted by C_1_ to C_10_. The mass difference between neighbouring peaks of 457 ± 18 Da corresponds to the molecular weight of a single chain. This signal is attributed to the clustering of fluoroalkylsulfanyl chains in the expanding plume. The C_1_ chain is abundant during synthesis and was not eliminated.

The result of a similar experiment with library **2** is shown in Fig.3(b). While low-mass fragments also exist here, we have only zoomed into the high-mass spectrum to better resolve its structure. The number of chains varies from 34 to 45, with small peaks up to around *n* = 50. Individual peaks can still be clearly resolved up to 25 000 Da. The characteristic distribution after laser desorption/photoionization is similar to MALDI measurements immediately after synthesis.

## Slow neutral beams of high-mass molecules

Slow molecules are essential for many experiments, such as beam deflection[31,32] or advanced matter-wave studies. Because state-of-the-art interferometers are capable of operating with de Broglie wavelengths down to *λ*_dB_ = 200 fm, we are aiming for neutral particle beams with a momentum of less than 25 000 Da × 40 m/s.

**Figure 4:**
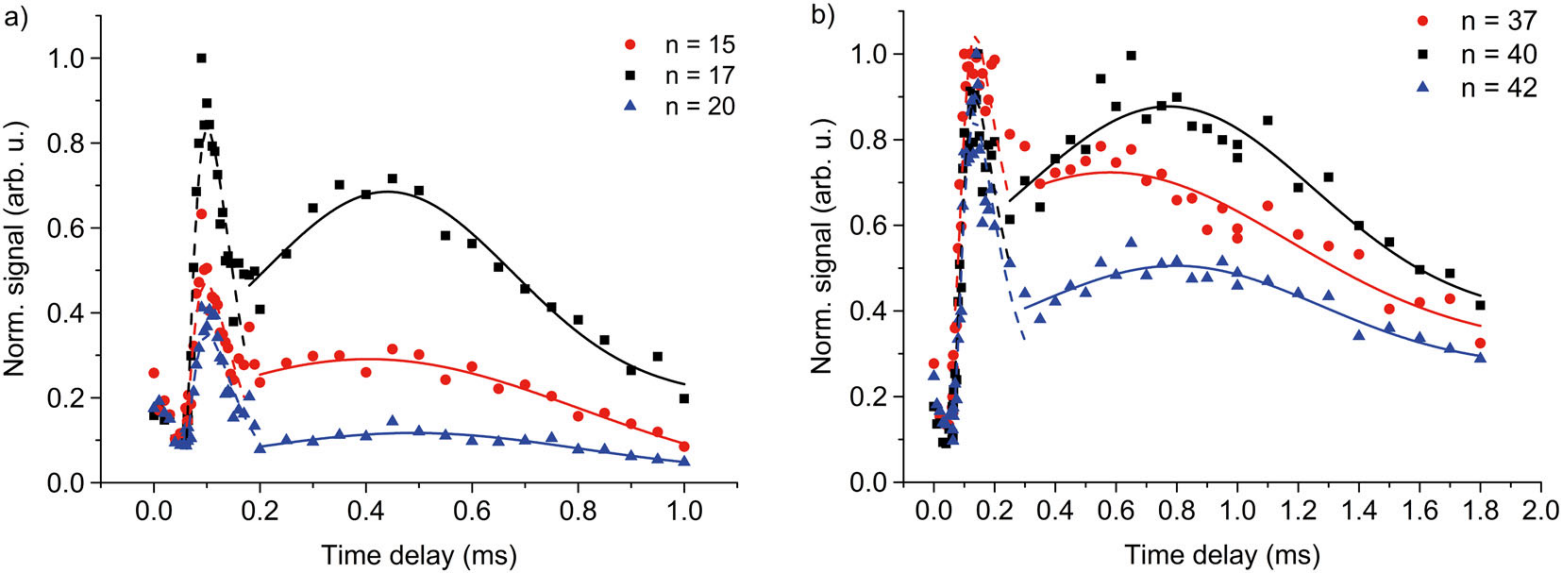
Time-of-flight distributions of some elements of library 1 (a) and library 2 (b). A delay time of 1 ms corresponds to a velocity of 39 m/s. Direct ions are seen as a precursor peak for flight times up to 200 µs. The continuous lines are Gaussian fits to the data. We find a most probable velocity of 45 ± 2 m/s for the *n* = 40 element of library 2. The signal drops by 50% at *v* ≃ 20 m/s.

Here, we measure the molecular velocity distribution by varying the delay time between the desorbing and the ionizing laser pulse. Figure4 shows data for the molecular libraries **1** and **2**. For a velocity analysis, we select three of the most prominent mass peaks of Fig.3. Our data show a bimodal velocity distribution: the fast component, best fitted by a Maxwell–Boltzmann distribution (dashed lines), is attributed to ions that are created in the source. The slow component (solid lines) represents neutral molecules that can only be detected if the VUV laser is on.

For library **1**, the neutral part of the distribution is best fitted with a Gaussian distribution centred at around *v* ≃ 90 m/s. There is still a substantial signal at 40 m/s (Fig.4(a)). Library elements of higher mass arrive with slightly lower speed. The slow fraction of library **2** (Fig.4(b)) is centred at around *v* = 45 m/s extending to velocities around 20 m/s for the most abundant mass peak. The functionalized pentaporphyrins are thus considerably more massive and substantially slower than similar macromolecular beams in earlier experiments.[33,34]

## Thermal robustness and photostability

The mass spectra and velocity curves of Figs3 and 4 prove already the feasibility of neutral laser desorption and VUV photoionization of even the most massive elements of both libraries. Here, we are additionally interested in the stability of these compounds under thermal load and VUV exposure.

Even though the measured velocities are low on an absolute scale in molecular beam physics, the most probable kinetic energy of the 22 000 Da particle at 45 m/s corresponds to 0.24 eV. If the molecular beam were thermal, the corresponding temperature of about 2700 K would certainly disintegrate all particles. However, Fig.4 indicates already that the desorption is not purely thermal.

**Figure 5:**
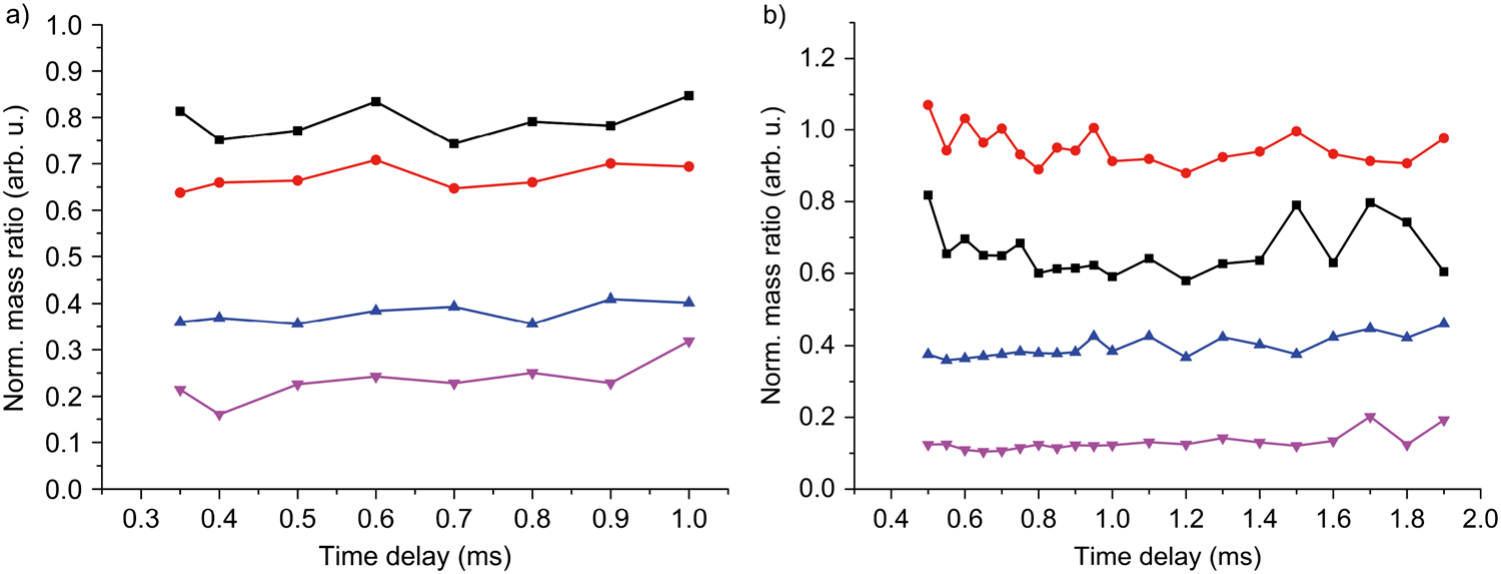
Evolution of the mass peaks in library 1 (a) and library 2 (b) as a function of the waiting time between laser desorption and post-ionization (all parameters as in Fig.2): *n* = 16, *n* = 36 (black squares), *n* = 19, *n* = 38 (red circles), *n* = 20, *n* = 42 (blue triangles), *n* = 21 and *n* = 44 (pink inverted triangles). All mass peaks are normalized to the signal of the most abundant mass (*n* = 17 in library 1, *n* = 40 in library 2).

In Fig.5, we search for indications of thermal fragmentation between desorption and detection, by analyzing the evolution of the molecular mass ratios in library **1** and library **2** as a function of the post-desorption time. All peak heights are normalized to the most prominent mass peak at *n* = 17 (library **1**) and *n* = 40 (library **2**). The absence of any significant deviation over time suggests that library **1** is stable over its transit time to the detector. The same is true for library **2**, in spite of its double number of chains. Even if there was some fragmentation in free flight – which we do not see in Fig.5 – matter-wave interferometry provides a way to distinguish fragmentation in the source from dissociation in the detector, as has been recently demonstrated.[5]

**Figure 6:**
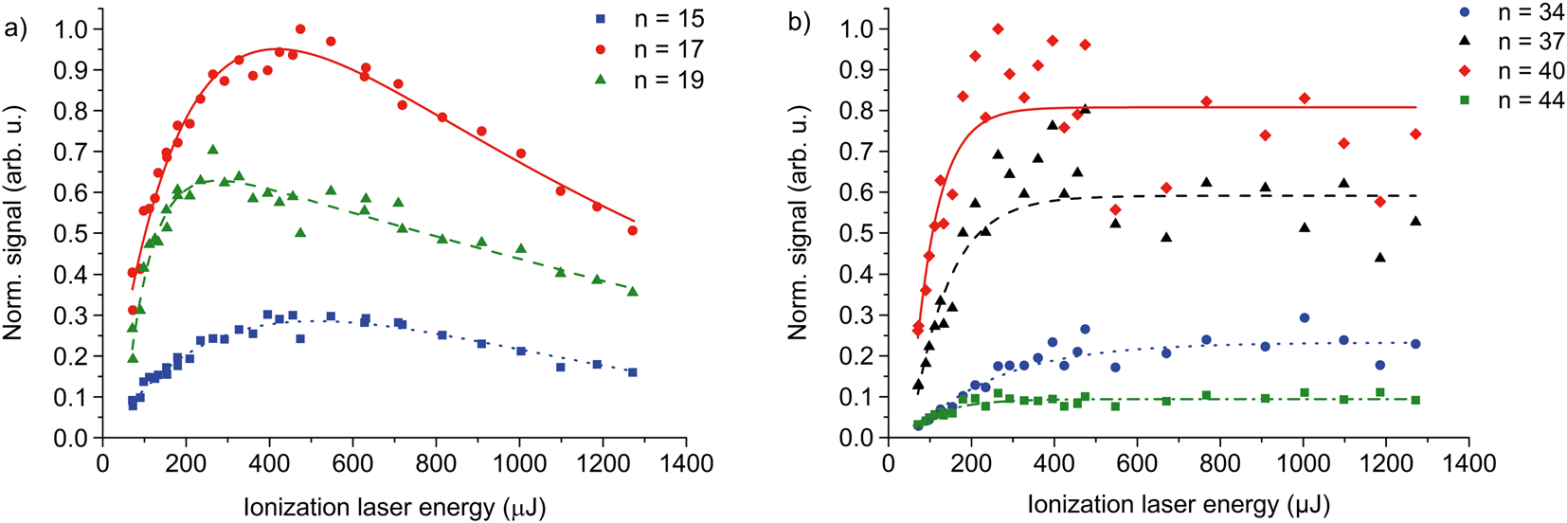
Photoionization signal *versus* laser energy of library 1 (a) and library 2 (b). The distinct symbols describe different numbers of fluoroalkylsulfanyl chains attached to the porphyrin core. The lines are drawn to guide the eye.[38] The signals were taken for a delay of 400 (library 1) and 800 µs (library 2) between desorption and ionization laser pulse.

Figure6 shows the signals of selected library members as a function of the ionizing laser energy. In both cases, the ion yield increases with growing pulse energy up to a saturation point. This corroborates the design assumption that the VUV ionization cross section of these molecules can be made sufficiently large by starting from an oligoporphyrin. The signal decrease with higher energy (library **1**) is attributed to photoinduced fragmentation. Interestingly, this is not observed for library **2**, because it has a larger number of fluoroalkylsulfanyl chains and more relaxation channels.[35] Thus, it is expected that the excess energy is dissipated to more constituents.[36,37]

## Conclusions

We have studied the volatilization and post-ionization properties of two new molecular libraries with different core conjugation but similar chains. Both can be efficiently launched at low velocities and photoionized at a laser wavelength of 157 nm even at masses up to 25 000 Da. For the most abundant library element, we still find a substantial signal of neutral particles with velocities *v* < 45 m/s. This matches the initial de Broglie wave requirement *λ*_dB_ ≥ 200 fm. Fluoroalkylsulfanyl-functionalized porphyrins therefore fulfil important expectations for future matter-wave studies. The synthesis of such compounds is demanding, but the concept of fluoroalkylsulfanyl-functionalized particles has certainly not yet reached its limits. It will be intriguing to explore ways to launch and detect neutral mass-specified particles even more complex than those presented here.

## References

[1] Juffmann T, Ulbricht H, Arndt M (2013). Experimental methods of molecular matter-wave optics. Rep. Prog. Phys.

[2] Hornberger K, Gerlich S, Haslinger P, Nimmrichter S, Arndt M (2012). Colloquium: Quantum interference of clusters and molecules. Rev. Mod. Phys.

[3] Eibenberger S, Gerlich S, Arndt M, Mayor M, Tüxen J (2013). Matter-wave interference of particles selected from a molecular library with masses exceeding 10 000 amu. Phys. Chem. Chem. Phys.

[4] Haslinger P, Dörre N, Geyer P, Rodewald J, Nimmrichter S, Arndt M (2013). A universal matter-wave interferometer with optical ionization gratings in the time domain. Nature Phys.

[5] Gerlich S, Gring M, Ulbricht H, Hornberger K, Tüxen J, Mayor M, Arndt M (2008). Matter-wave metrology as a complementary tool for mass spectrometry. Angew. Chem. Int. Ed. Engl.

[6] Gring M, Gerlich S, Eibenberger S, Nimmrichter S, Berrada T, Arndt M, Ulbricht H, Hornberger K, Müri M, Mayor M, Böckmann M, Doltsinis NL (2010). Influence of conformational molecular dynamics on matter wave interferometry. Phys. Rev. A.

[7] Eibenberger S, Gerlich S, Arndt M, Tüxen J, Mayor M (2011). Electric moments in molecule interferometry. New J. Phys.

[8] Eibenberger S, Cheng X, Cotter JP, Arndt M (2014). Absolute absorption cross sections from photon recoil in a matter-wave interferometer. Phys. Rev. Lett.

[9] Fenn JB, Mann M, Meng CK, Wong SF, Whitehouse CM (1989). Electrospray ionization for mass spectrometry of large biomolecules. Science.

[10] Tanaka K, Waki H, Ido Y, Akita S, Yoshida Y, Yoshida T, Matsuo T (1988). Protein and polymer analyses up to m/z 100 000 by laser ionization time-of-flight mass spectrometry. Rapid Commun. Mass Spectrom.

[11] Cai Y, Peng W-P, Kuo S-J, Sabu S, Han C-C, Chang H-C (2002). Optical detection and charge-state analysis of MALDI-generated particles with molecular masses larger than 5 MDa. Anal. Chem.

[12] Breuker K, Knochenmuss R, Zhang J, Stortelder A, Zenobi R (2003). Thermodynamic control of final ion distributions in MALDI: in-plume proton transfer reactions. Int. J. Mass Spectrom.

[13] Glückmann M, Karas M (1999). The initial ion velocity and its dependence on matrix, analyte and preparation method in ultraviolet matrix-assisted laser desorption/ionization. J. Mass Spectrom.

[14] Tomalová I, Frankevich V, Zenobi R (2014). On initial ion velocities in MALDI: A novel FT-ICR MS approach. Int. J. Mass Spectrom.

[15] Grotemeyer J, Boesl U, Walter K, Schlag EW (1986). Biomolecules in the Gasphase. II. Multiphoton Ionization Mass Spectrometry of Angiotensin I. OMS Lett.

[16] Grotemeyer J, Bosel U, Walter K, Schlag EW (1986). Biomolecules in the Gas Phase. 1. Multiphoton-Ionization Mass Spectrometry of Native Chlorophylls. J. Am. Chem. Soc.

[17] Schlag E, Grotemeyer J, Levine R (1992). Do large molecules ionize?. Chem. Phys. Letts.

[18] Marksteiner M, Haslinger P, Sclafani M, Ulbricht H, Arndt M (2009). UV and VUV ionization of organic molecules, clusters, and complexes. J. Phys. Chem. A.

[19] Marksteiner M, Haslinger P, Ulbricht H, Sclafani M, Oberhofer H, Dellago C, Arndt M (2008). Gas-phase formation of large neutral alkaline-earth metal tryptophan complexes. J. Am. Soc. Mass Spectrom.

[20] Akhmetov A, Moore JF, Gasper GL, Koin PJ, Hanley L (2010). Laser desorption postionization for imaging MS of biological material. J. Mass Spectrom.

[21] Hanley L, Zimmermann R (2009). Light and Molecular Ions: The Emergence of Vacuum UV Single-Photon Ionization in MS. Anal. Chem.

[22] Gasper GL, Carlson R, Akhmetov A, Moore JF, Hanley L (2008). Laser desorption 7.87 eV postionization mass spectrometry of antibiotics in Staphylococcus epidermidis bacterial biofilms. Proteomics.

[23] Edirisinghe P, Moore J, Calaway W, Veryovkin I, Igor V, Pellin M, Hanley L (2006). Vacuum ultraviolet postionization of aromatic groups covalently bound topeptides. Anal. Chem.

[24] Hanley L, Edirisinghe PD, Calaway WF, Veryovkin IV, Pellin MJ, Moore JF (2006). 7.87 eV postionization of peptides containing tryptophan or derivatized with fluorescein. Appl. Surf. Sci.

[25] Tüxen J, Eibenberger S, Gerlich S, Arndt M, Mayor M (2011). Highly Fluorous Porphyrins as Model Compounds for Molecule Interferometry. Eur. J. Org. Chem.

[26] Schmid P, Stöhr F, Arndt M, Tüxen J, Mayor M (2013). Single-Photon Ionization of Organic Molecules Beyond 10 kDa. J. Am. Soc. Mass Spectrom.

[27] Felix L, Sezer U, Arndt M, Mayor M (2014). Synthesis of Highly Fluoroalkyl-Functionalized Oligoporphyrin Systems. Eur. J. Org. Chem.

[28] Khandelwal SC, Roebber JL (1975). The photoelectron spectra of tetraphenylporphine and some metallotetraphenylporphyrins. Chem. Phys. Letts.

[29] Dolgounitcheva O, Zakrzewski VG, Ortiz JV (2005). Ab Initio Electron Propagator Calculations on the Ionization Energies of Free Base Porphine, Magnesium Porphyrin, and Zinc Porphyrin. J. Phys. Chem. A.

[30] Wiley WC, McLaren IH (1955). Time-of-flight mass spectrometer with improved resolution. Rev. Sci. Instrum.

[31] Heiles S, Schäfer R (2014). Dielectric Properties of Isolated Clusters Beam Deflection Studies.

[32] Heer WAD, Kresin VV, Sattler KD (2011). Handbook of Nanophysics.

[33] Maxwell S, Brahms N, DeCarvalho R, Glenn D, Helton J, Nguyen S, Patterson D, Petricka J, DeMille D, Doyle J (2005). High-Flux Beam Source for Cold, Slow Atoms or Molecules. Phy. Rev. Lett.

[34] Patterson D, Tsikata E, Doyle JM (2010). Cooling and collisions of large gas phase molecules. Phys. Chem. Chem. Phys.

[35] Hu Y, Hadas B, Davidovitz M, Balta B, Lifshitz C (2003). Does IVR take place prior to peptide ion dissociation?. J. Phys. Chem. A.

[36] Brédas J-L, Beljonne D, Coropceanu V, Cornil J (2004). Charge-transfer and energy-transfer processes in π-conjugated oligomers and polymers: a molecular picture. Chem. Rev.

[37] Devadoss C, Bharathi P, Moore JS (1996). Energy Transfer in Dendritic Macromolecules:  Molecular Size Effects and the Role of an Energy Gradient. J. Am. Chem. Soc.

[38] Wahl M, Wucher A (1994). VUV photoionization of sputtered neutral silver clusters. Nucl. Instr. Meth. Phys. Res.B.

